# E3 Ubiquitin Ligases in Neurodevelopmental Disorders

**DOI:** 10.3390/cells15181703

**Published:** 2026-09-19

**Authors:** Shuwan Wang, Xiang Pan, Kang Zhang, Deqiang Zhao, Xiaoxiao Chen, Yibei Wang, Haixia Hu, Yanfeng Zhang

**Affiliations:** 1 School of Exercise and Health, Shanghai University of Sport, Shanghai 200438, China; shuwan_wang0705@163.com; 2 China Institute of Sport Science, Beijing 100061, China; 3 Graduate School of Health and Sports Science, Juntendo University, Inzai 270-1695, Japan; 4 School of Physical Education and Sports, Sichuan Normal University, Chengdu 610066, China; 5 School of Physical Education and Sports Rehabilitation, Jinzhou Medical University, Jinzhou 121001, China; 6 Aiyoudong Children and Youth Sports Health Research Institute, Weifang 261041, China

**Keywords:** neurodevelopmental disorders, E3 ubiquitin ligases, ubiquitination, protein homeostasis, neurogenesis, synaptic development

## Abstract

**Highlights:**

**What are the main findings?**

• E3-related neurodevelopmental disorders are not limited to single E3 ubiquitin ligases but also involve E3 complex components, substrate recognition factors, and other regulatory proteins associated with ubiquitination; genetic abnormalities in different genes entail distinct genetic and functional consequences.
• GO enrichment analysis and mechanistic evidence indicate that various E3-related abnormalities exhibit functional convergence in specific neurodevelopmental and cellular processes; however, the stages of therapeutic translation for these mechanisms differ significantly.

**What are the implications of the main findings?**

• *UBE3A*-related Angelman syndrome currently offers the clearest mechanism-based therapeutic pathway, whereas research into most non-*UBE3A* mechanisms remains at the stage of preclinical pathway analysis or candidate substrate validation.
• Future studies need to further elucidate the specific functional consequences of various pathogenic variants, validate disease-relevant direct substrates and reproducible functional biomarkers, and consider factors such as developmental stage, dosage, and cell type in therapeutic development.

**Abstract:**

Neurodevelopmental disorders (NDDs) comprise a heterogeneous group of conditions that may present with developmental delay, intellectual disability, language impairment, epilepsy, autism-like features, motor abnormalities, or syndromic manifestations. Ubiquitination is an essential post-translational mechanism that regulates protein stability, localization, trafficking, and signaling. Because E3 ubiquitin ligases determine substrate specificity in this process, abnormalities in E3 ligases or E3-associated complexes can selectively affect proteins required for brain development and neuronal function. This review summarizes genetic and functional evidence linking E3 ubiquitin ligases, E3-complex components, substrate-recognition factors, and related ubiquitination regulators to NDDs. Gene Ontology enrichment analysis and mechanistic studies suggest that these genes converge on several major biological themes, including early neurodevelopment, neuronal migration, synaptic and circuit maturation, proteostasis, metabolism, and intracellular trafficking. However, the strength and translational relevance of the available evidence vary across genes. *UBE3A*-related Angelman syndrome currently provides the clearest example of mechanism-guided therapeutic development through *UBE3A* restoration and paternal allele reactivation. In contrast, many non-*UBE3A* mechanisms remain at the stage of pathway analysis, preclinical modulation, or candidate substrate validation. Future studies should clarify variant-specific functional effects, define direct disease-relevant substrates, and establish reproducible readouts that can support mechanism-based therapeutic development.

## 1. Introduction

Neurodevelopmental disorders (NDDs) originate during early brain development and often present with developmental delay (DD), intellectual disability (ID), language impairment, autism-like behaviors, epilepsy, motor abnormalities, or deficits in learning and memory. Although individual NDDs differ clinically, many converge on disrupted neural progenitor proliferation and differentiation, neuronal migration, axonal and dendritic development, synapse formation, synaptic plasticity, and neural network maturation [1,2]. Large-scale exome and genome sequencing studies have identified rare pathogenic or risk-associated variants, especially de novo and loss-of-function variants, in severe developmental disorders and autism spectrum disorder (ASD) [3,4]. These findings suggest that NDDs often involve disruptions in multiple developmental pathways rather than isolated molecular abnormalities [5].

Ubiquitination is a post-translational modification that controls protein stability, localization, activity, trafficking, and signaling. The canonical ubiquitination cascade is mediated by E1 ubiquitin-activating enzymes, E2 ubiquitin-conjugating enzymes, and E3 ligases [6]. Among these enzymes, E3 ligases provide substrate recognition and largely determine ubiquitination specificity [7]. E3 dysfunction therefore does not necessarily produce generalized proteasomal failure. Instead, it can selectively alter the abundance, localization, or function of proteins that are important for neural development and neuronal function. This selectivity is particularly relevant in neurons, where protein turnover and trafficking must be tightly regulated across spatially separated cellular compartments [7,8]. Human genetic studies have now linked a growing range of E3 ligases, E3-complex components, substrate-recognition factors, and related ubiquitination regulators to NDDs [3,9].

As the genetic landscape of E3-related NDDs continues to expand, the key question is no longer simply to identify additional disease-associated genes but to determine whether these genes share functional links that can be integrated into a broader mechanistic framework. This review focuses on E3 ligases, E3-complex components, substrate-recognition factors, and related ubiquitination regulators that have relatively clear human genetic support and at least some functional evidence. At present, only a few well-established genes have accumulated relatively complete genetic, functional, and disease-model evidence, with some directions beginning to move toward therapeutic translation. In contrast, many more recently expanded E3-related genes remain largely at the level of disease association, candidate substrates, or localized mechanistic observations. These findings remain fragmented, and genetic associations, functional mechanisms, and therapeutic relevance have not yet been connected within a unified framework. Therefore, this review not only broadens the genetic scope of E3-related NDDs but also integrates Gene Ontology enrichment analysis with mechanism-focused studies to summarize functional convergence across neurodevelopmental and cellular processes. It further distinguishes mechanisms that have developed relatively clear therapeutic paths, pathways with preclinical modulatory evidence, and candidate substrates or functional readouts that still require further validation.

## 2. NDD-Associated E3 Ligases and E3-Complex Components

**Figure 1:**
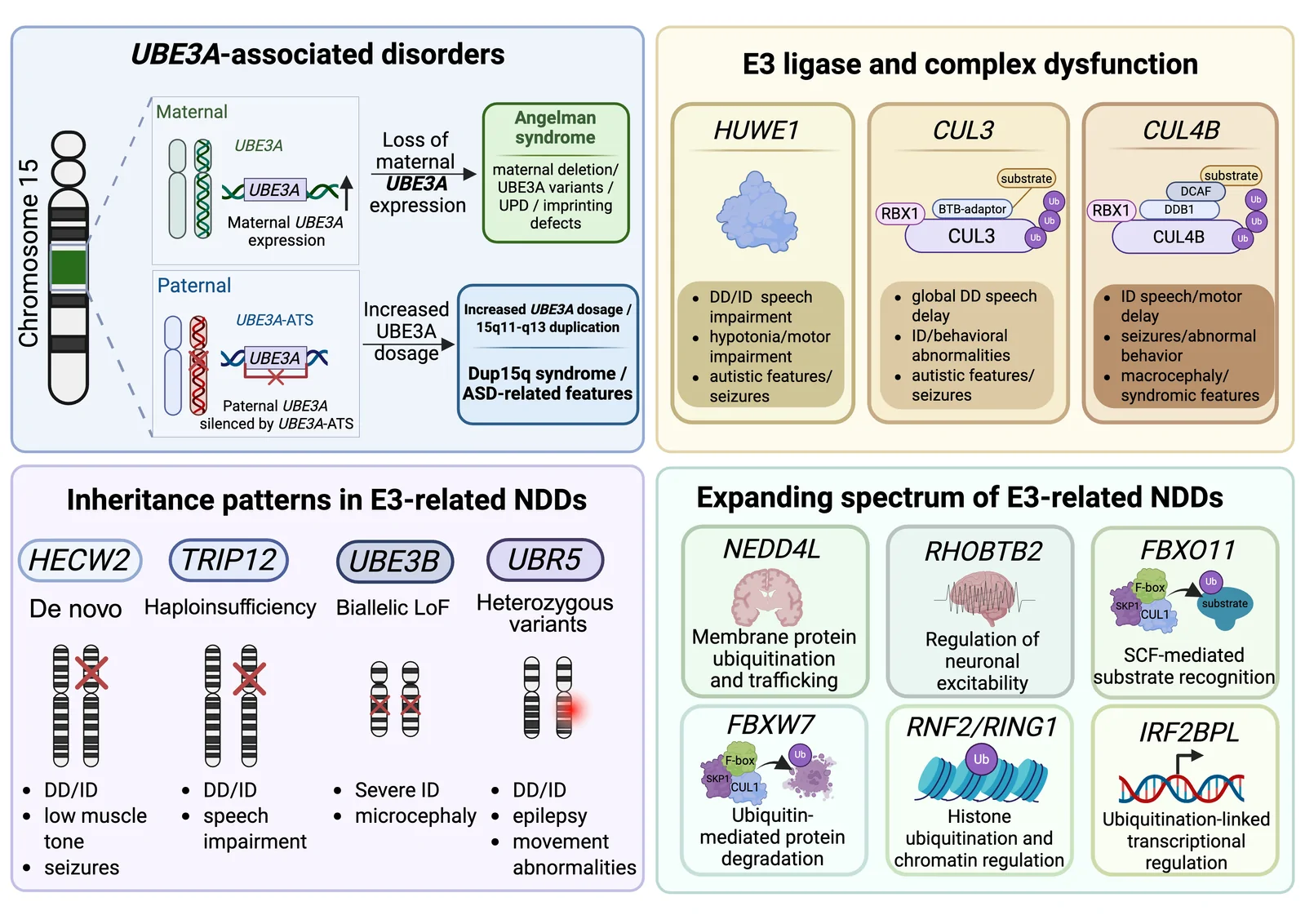
Genetic landscape of representative E3-related neurodevelopmental disorders.Genetic landscape of representative E3-related neurodevelopmental disorders. Upward and downward arrows indicate increased and decreased expression or dosage, respectively; red crosses indicate silencing or loss of function.

Genetic studies have shown that NDDs have a highly heterogeneous genetic architecture, in which both de novo and inherited variants can affect multiple genes involved in neurodevelopment [10]. E3 ubiquitin ligases and E3-complex components represent one group of molecular nodes of particular interest, with abnormalities reported in developmental delay, intellectual disability, epilepsy, ASD-related features, language impairment, motor abnormalities, and various syndromic developmental disorders [11,12]. E3-related NDD genes do not constitute a single disease entity, nor can they be clinically classified simply according to E3 ligase family. Different genes vary in inheritance pattern, variant type, dosage sensitivity, substrate recognition, complex assembly, and the neurodevelopmental processes in which they act [13,14]. In this context, *UBE3A* represents the clearest example involving genomic imprinting, dosage sensitivity, and translational research [15]. *HUWE1*, *CUL3*, *CUL4B*, *HECW2*, *TRIP12*, *UBE3B*, and *UBR5* illustrate the recurrent involvement of E3 dysfunction across different NDD phenotypes. The inclusion of *NEDD4L*, *RHOBTB2*, *FBXO11*, *FBXW7*, *RNF2/RING1*, and *IRF2BPL* further expands this disease spectrum beyond a small number of classic syndromic genes, suggesting that ubiquitination-related pathways may contribute to NDD pathogenesis through multiple levels, including neuronal excitability, cell-fate regulation, chromatin state, and transcriptional homeostasis (Figure 1). Other E3 ligases, E3-complex components, substrate adaptors, and ubiquitination-associated regulators associated with neurodevelopmental phenotypes are summarized in Table 1.

### 2.1. UBE3A

*UBE3A* encodes E6-associated protein (E6AP), a HECT-type E3 ubiquitin ligase and one of the most extensively characterized E3-related genes associated with NDDs. Patients with Angelman syndrome (AS, OMIM #105830) typically exhibit severe DD, ID, markedly limited speech, ataxia, seizures, sleep abnormalities, and characteristic behavioral phenotypes [16,17,18]. Motor, language, and daily-living impairments can persist across age groups [19]. Autism-like features, sleep disturbance, and challenging behaviors are also reported in AS, but these features should be interpreted within the broader context of global DD and syndromic neurodevelopmental impairment [20,21].

Most cases of AS are associated with maternal 15q11-q13 deletion, pathogenic *UBE3A* variants, paternal uniparental disomy, or imprinting defects [17,22]. These changes reduce or abolish maternal *UBE3A* function in neurons. In mature neurons, paternal *UBE3A* is usually silenced by *UBE3A-ATS*-related antisense transcription, making neuronal function dependent on the maternal allele. This allele-specific pattern also provides the rationale for paternal *UBE3A* reactivation therapy [23,24,25]. Increased *UBE3A* dosage, by contrast, is associated with 15q11-q13 duplication syndrome (OMIM #608636), ASD-like phenotypes, and sleep EEG abnormalities [26,27,28,29,30]. *UBE3A* therefore represents a key example for understanding E3-related NDD mechanisms; however, its neuronal imprinting and dosage sensitivity are relatively unique features that may not fully represent other E3-related disorders.

### 2.2. HUWE1, CUL3, and CUL4B

*HUWE1* is located at Xp11.22 and encodes a large HECT-type E3 ubiquitin ligase. Unlike the relatively distinctive neuronal imprinting mechanism in *UBE3A*-related disorders, *HUWE1*-related NDDs more prominently illustrate the effects of X-linked inheritance, gene–dosage alteration, and pathogenic sequence variants on neurodevelopment. Whole-gene duplications and copy-number gains involving *HUWE1* can cause X-linked intellectual disability [31], and pathogenic sequence variants have also been associated with *HUWE1*-related neurodevelopmental disorder [32,33]. The clinical presentation is mainly characterized by developmental delay, intellectual disability, and speech impairment, while some patients may also present with hypotonia, motor impairment, autistic features, seizures, and variable dysmorphic, growth-related, or feeding-related abnormalities. Functional evidence suggests that *HUWE1* may participate in neuronal precursor proliferation, differentiation, apoptosis, and neuronal maturation, with N-Myc- and p53-related pathways providing mechanistic links to neurodevelopmental processes [34,35]. *HUWE1*-mediated TTBK2 degradation further connects this ligase to primary-cilium dynamics and cerebellar granule neuron progenitor differentiation [36]. Thus, *HUWE1* is better viewed as a representative example of dosage- and function-sensitive HECT-type E3 dysfunction in X-linked NDDs, rather than as a simple extension of the *UBE3A* imprinting model.

*CUL3* encodes a scaffold component of Cullin-RING E3 ligase complexes, and its pathogenic mode differs from that of substrate-specific HECT ligases such as *HUWE1*. Heterozygous *CUL3* variants, particularly de novo loss-of-function variants [37,38,39] or variants that affect *CUL3*-BTB adaptor interaction [40], have been associated with neurodevelopmental disorder with or without autism or seizures (NEDAUS; OMIM #619239). Patients typically present with global developmental delay, speech delay, intellectual impairment, and behavioral abnormalities, and some cases also show autistic features or seizures. Because *CUL3*-containing CRL complexes rely on different adaptor proteins for substrate recruitment, the effects of *CUL3* variants are unlikely to be restricted to a single substrate and may instead disturb multiple downstream pathways [41]. Consistent with this, functional evidence has linked *CUL3* to cortical neurogenesis, cytoskeletal regulation, neuronal migration, and protein homeostasis, although these mechanisms are more appropriately discussed together in Section 3 [42,43].

*CUL4B* encodes another Cullin-RING E3 ligase scaffold/core component and is part of the CRL4B complex. Pathogenic *CUL4B* variants can cause X-linked intellectual developmental disorder, Cabezas type (OMIM #300354) [44,45]. Patients commonly present with intellectual disability, speech and motor delay, seizures, and abnormal behavior, and may also show relative macrocephaly, central obesity, hypogonadism, pes cavus, tremor, and dysmorphic or congenital features. Similar to *CUL3*, the pathogenic relevance of *CUL4B* lies not only in abnormalities of individual substrates, but also in altered CRL complex-dependent regulation. *CUL4B* has been linked to chromatin-associated regulation and neuronal gene-expression control [46], while more recent functional work has connected *CUL4B* mutations or deficiency to impaired cortical neurogenesis [47] and synaptic dysfunction [48]. A further mechanistic study identified GABA transporter 1 as a downstream target relevant to *CUL4B* mutation-associated epilepsy, providing a more specific link between CRL4B dysfunction and inhibitory synaptic transmission [49]. Together, *CUL3* and *CUL4B* extend E3-related NDDs beyond dosage or protein-function changes in individual E3 ligases to abnormalities in E3-complex assembly, substrate recruitment, and complex-dependent gene regulation.

### 2.3. HECW2, TRIP12, UBE3B, and UBR5

*HECW2* and *TRIP12* both encode HECT-type E3 ubiquitin ligases, further extending the HECT-type E3-related NDD spectrum beyond *UBE3A* and *HUWE1*. De novo pathogenic variants in *HECW2* have been associated with neurodevelopmental delay, intellectual disability, hypotonia, and seizures, with some patients also showing abnormal movements, feeding problems, language impairment, behavioral abnormalities, or dysmorphic features [50,51,52]. Functionally, *HECW2* may be linked to neuronal differentiation and neurogenesis through p73-related neurodevelopmental regulation [50,53]. Pathogenic variants or haploinsufficiency of *TRIP12* are associated with Clark–Baraitser syndrome (CLABARS; OMIM #617752) or *TRIP12*-related neurodevelopmental disorder. The clinical presentation is mainly characterized by developmental delay, intellectual disability, and speech impairment and may also include autistic features, behavioral problems, obesity tendency, or characteristic facial features [54,55,56]. Unlike the imprinting-centered model of *UBE3A*, *HECW2* and *TRIP12* indicate that HECT-type E3 ligases can enter the NDD spectrum through de novo variants, haploinsufficiency, and distinct patient-level phenotypes.

*UBE3B* encodes a HECT-type E3 ubiquitin ligase, and its associated disorder more prominently reflects an autosomal recessive/biallelic loss-of-function mechanism. Biallelic pathogenic variants in *UBE3B* can cause Kaufman oculocerebrofacial syndrome (KOS; OMIM #244450), also described as blepharophimosis-ptosis-intellectual-disability syndrome [57,58]. Patients commonly present with developmental delay, severe intellectual disability, microcephaly, hypotonia, growth retardation, ocular and facial anomalies, congenital anomalies, and hypocholesterolemia-related features, with subsequent cases further expanding the phenotypic spectrum of this syndrome [59,60]. Taken together, these findings indicate that *UBE3B* extends E3-related NDDs to biallelic loss-of-function and syndromic developmental phenotypes, in contrast to several genes discussed above that more often involve dominant or de novo variants. Functional studies more directly link *UBE3B* deficiency to neuronal morphology and synaptic development, including altered synapse number through *Ube3b*-dependent regulation of *Ppp3cc* [61].

*UBR5* encodes a HECT-type E3 ubiquitin ligase with N-recognin-related functions. Available case-level and cohort-level evidence has linked heterozygous pathogenic *UBR5* variants, especially loss-of-function or functionally disruptive variants, to a neurodevelopmental syndrome characterized by developmental delay, intellectual disability, speech/language impairment, autistic features, behavioral abnormalities, epilepsy, movement disorders, and variable congenital or syndromic features [62,63]. Compared with *HECW2*, TRIP12, and *UBE3B*, the disease boundaries of *UBR5*-related NDD are still being defined through case-level and cohort-level data. At present, it is therefore better understood as an expanding E3-related NDD association. Functionally, *UBR5* may connect protein turnover, transcriptional regulation, cell-cycle control, and developmental signaling. The relationship between *UBR5*/UBR7 and Notch signaling provides a clue to its role in developmental regulation, although this should not be expanded into a full mechanistic pathway in this section [64]. Together, these genes further broaden the genetic and clinical scope of E3-related NDDs and provide a transition to the subsequent discussion of specific mechanisms organized by neurodevelopmental process.

### 2.4. NEDD4L, RHOBTB2, FBXO11, FBXW7, RNF2/RING1, and IRF2BPL

*NEDD4L* encodes a HECT-type E3 ubiquitin ligase, and pathogenic variants in this gene have been linked to periventricular nodular heterotopia 7 (PVNH7; OMIM #617201) and related neurodevelopmental abnormalities [65]. Patients mainly present with developmental delay, intellectual disability, epilepsy, and cortical malformations, and some cases may also show language, motor, or tone abnormalities, as well as syndromic features such as cleft palate and syndactyly [66]. The relationship of *NEDD4L* with AKT-mTOR signaling, neuronal migration, and cortical development further provides a functional clue for the cortical malformations and epilepsy observed in some patients [65]. *RHOBTB2* encodes an atypical Rho GTPase/BTB-domain protein with a functional background related to *CUL3*-dependent ubiquitination [67]. Pathogenic *RHOBTB2* variants have been associated with developmental and epileptic encephalopathy 64 (DEE64; OMIM #618004) and a broader spectrum of *RHOBTB2*-associated neurodevelopmental disorders. Patients commonly present with early-onset epilepsy, developmental delay, intellectual disability, movement disorders, and tone abnormalities [67,68]. The effects of variant location on protein stability and neuronal excitability further suggest that *RHOBTB2*-related disorders may involve abnormalities in protein homeostasis and neural-network excitability [69].

*FBXO11* and *FBXW7* both encode F-box proteins that participate in substrate recognition within SCF-type Cullin-RING E3 ligase complexes. Pathogenic *FBXO11* variants are associated with intellectual developmental disorder with dysmorphic facies and behavioral abnormalities (IDDFBA; OMIM #618089), which is mainly characterized by developmental delay, intellectual disability, limited speech development, and behavioral abnormalities, with some patients also showing autistic features, hypotonia, dysmorphic facial features, epilepsy, or growth abnormalities [70]. The effects of *FBXO11*-related variants on protein expression and subcellular localization provide functional support for its involvement in SCF-type E3 ligase-related neurodevelopmental abnormalities [70]. Pathogenic *FBXW7* variants are associated with a neurodevelopmental syndrome mainly characterized by global developmental delay, intellectual disability, hypotonia, and limited speech development, with some patients also presenting with epilepsy, macrocephaly, structural brain abnormalities, and other congenital anomalies [71,72]. As a substrate-recognition component, *FBXW7* can connect SCF complex dysfunction with cell-cycle control, cell-fate regulation, and neurodevelopmental phenotypes by influencing the degradation of specific proteins and the regulation of neurodevelopment-related signaling pathways [71]. These disease associations indicate that E3-related NDDs can arise not only from abnormalities in catalytic cores or scaffold components, but also from disruption at the level of substrate recognition.

*RNF2* and *RING1* encode Polycomb-associated RING-type E3 ligases and are components of PRC1, participating in chromatin-state and gene-expression regulation through histone H2A monoubiquitination [73]. De novo or pathogenic variants in *RNF2* or *RING1* have been linked to syndromic neurodevelopmental disorders, in which patients may present with developmental delay, intellectual disability, behavioral abnormalities, epilepsy, and variable facial or congenital anomalies [74,75,76]. The relationships of these variants with H2A ubiquitination, Polycomb chromatin binding, and neurogenesis make PRC1-related chromatin regulation an important clue connecting E3 dysfunction with neurodevelopmental abnormalities [75]. *IRF2BPL* encodes a protein with transcriptional regulatory and ubiquitination-related functions. Pathogenic variants in *IRF2BPL* can cause neurodevelopmental disorder with regression, abnormal movements, loss of speech, and seizures (NEDAMSS; OMIM #618088). In addition to developmental delay and intellectual disability, patients may gradually develop loss of speech, seizures, ataxia, movement disorders, and other neurodegenerative features [77,78]. Unlike the disease associations discussed above, which are mainly characterized by developmental delay, intellectual disability, or epilepsy, *IRF2BPL* also highlights neurodevelopmental regression and motor dysfunction, extending the mechanistic scope of E3-related NDDs to ubiquitination-linked transcriptional regulation and processes involved in neuronal maintenance.

## 3. Neurodevelopmental Processes and Specific Mechanisms Affected by E3 Ubiquitin Ligase Abnormalities

E3-related neurodevelopmental disorders do not arise from abnormalities in a single class of ubiquitination-related proteins. In addition to catalytically active E3 ubiquitin ligases, disease-related genes may encode E3-complex components, substrate-recognition factors, and other ubiquitination-associated regulatory proteins [9]. The clinical phenotypes associated with these genes may overlap, but phenotypic overlap does not necessarily imply a shared molecular mechanism. Therefore, after summarizing gene–disease associations, it is necessary to examine whether the relevant genes show convergence at the level of functional annotation and to interpret these functional clues in the context of mechanism-focused studies. Existing mechanistic evidence broadly falls into several recurrent neurodevelopmental and cellular processes, including early neurogenesis and cell-fate regulation, neuronal migration and cytoskeletal control, synaptic and neural-circuit maturation, as well as proteostasis, metabolic regulation, and cellular homeostasis.

### 3.1. Gene Ontology Enrichment Analysis of E3-Related Genes

**Figure 2:**
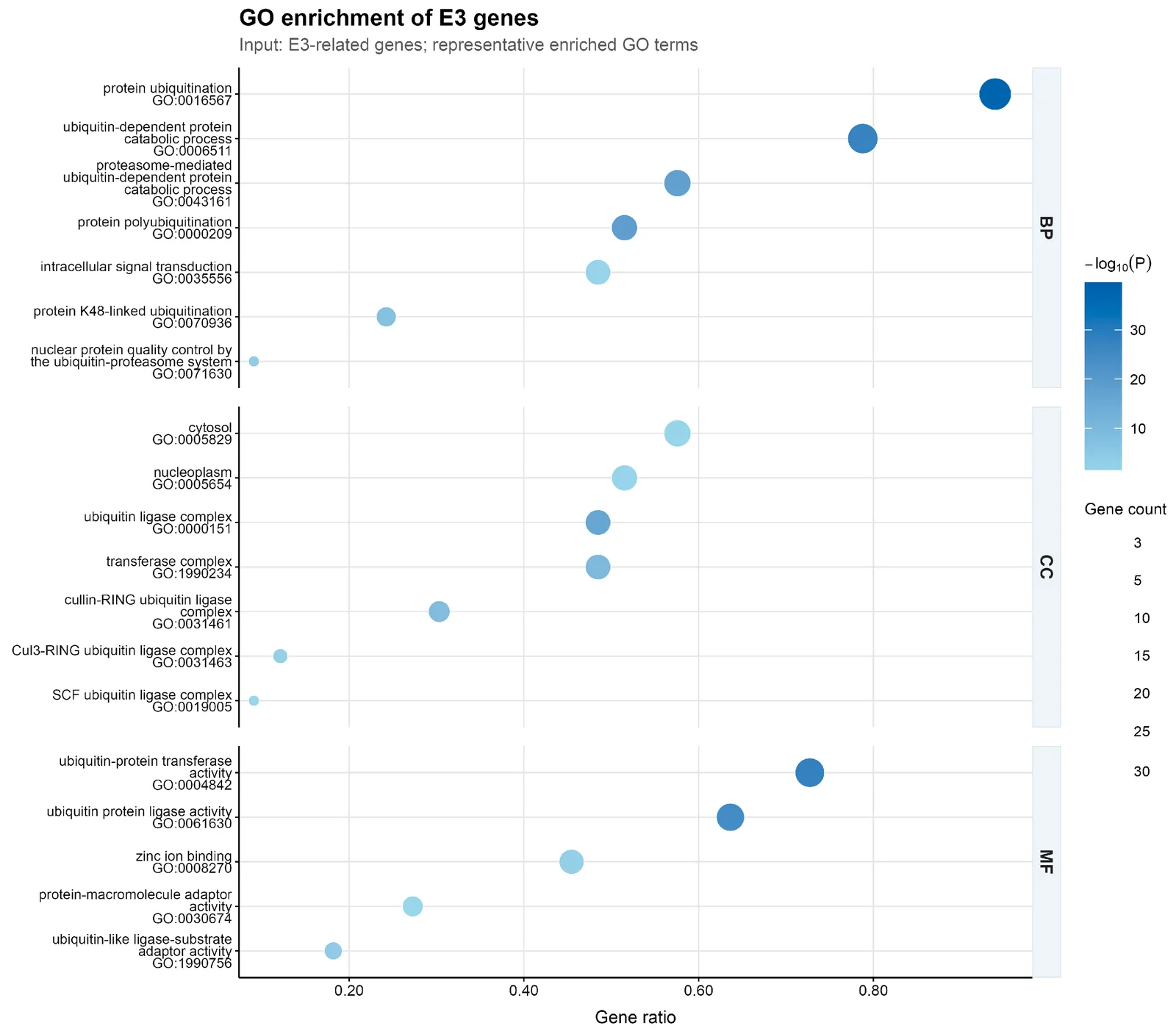
Gene Ontology enrichment profile of E3-related genes and mechanistic nodes discussed in this review.

To provide an overall functional view of the E3-related genes included in this review, we performed Gene Ontology (GO) enrichment analysis across biological process (BP), cellular component (CC), and molecular function (MF) categories. GO provides a structured framework for describing the biological processes, molecular activities, and cellular contexts associated with a gene set [79]. Enrichment analysis was performed using g:Profiler [80] (Figure 2).

The enrichment profile was dominated by ubiquitination and ubiquitin-dependent protein turnover, consistent with the shared molecular role of the genes included in the analysis. Within the BP category, protein ubiquitination showed the strongest enrichment, accompanied by terms related to ubiquitin-dependent and proteasome-mediated protein catabolism, polyubiquitination, and K48-linked ubiquitination. Together, these terms reflect successive components of the ubiquitin–proteasome system, from the attachment and extension of ubiquitin chains to the recognition and degradation of ubiquitinated proteins. The enrichment of nuclear protein quality control further suggests that these processes are not restricted to cytoplasmic protein turnover but also involve the maintenance of nuclear proteostasis. Intracellular signal transduction was also represented, consistent with the broader regulatory consequences that changes in protein abundance or stability can exert on cellular signaling. The CC and MF results provide complementary information about how these processes are organized. Enrichment of ubiquitin ligase complexes, including cullin-RING, *CUL3*-RING, and SCF complexes, reflects the contribution of multi-subunit E3 systems in addition to single-protein E3 ligases. This is particularly relevant to genes such as *CUL3* and other substrate-recognition or adaptor components, whose effects depend on complex assembly and substrate recruitment rather than on an isolated catalytic activity. Correspondingly, the MF category was enriched not only for ubiquitin-protein transferase and ubiquitin ligase activities, but also for substrate-adaptor and protein-adaptor functions. Thus, the enrichment pattern captures two related levels of E3 regulation: the catalytic transfer of ubiquitin and the molecular recognition or recruitment that determines which proteins are targeted.

Taken together, the GO results show that the genes considered here converge at the level of ubiquitination machinery, protein turnover, and E3-complex organization despite their genetic and clinical heterogeneity. These enrichments should not be interpreted as evidence that all genes act through the same substrates or neurodevelopmental pathways. Rather, they provide an annotation-level functional framework within which the gene-specific mechanisms can be considered. The corresponding genes, molecular modules, and experimentally supported neurodevelopmental effects are summarized in Table 2.

### 3.2. Neural Progenitor Proliferation, Differentiation, and Early Brain Development

Neural progenitor proliferation, cell-cycle exit, and neuronal differentiation are critical early developmental processes affected by several E3-related genes. *CUL4B* influences neural progenitor growth, cell-cycle regulation, and cortical neurogenesis [81]. *CUL4B* mutations impair human cortical neurogenesis through PP2A-dependent inhibition of AKT/ERK signaling [47], and *CUL4B*-based E3 complexes can participate in mitosis and brain development by recruiting phosphorylation-specific DCAFs [82]. These findings indicate that *CUL4B* abnormalities may affect brain development at the level of progenitor expansion, mitosis, and cortical neurogenesis before mature neuronal function is established.

*HUWE1* promotes N-Myc degradation, thereby contributing to the regulation of neural progenitor proliferation and neuronal differentiation during brain development [34], and can promote neurogenesis by suppressing the N-Myc-DLL3 cascade [83]. In models of *HUWE1*-associated ID, enhanced p53 signaling impairs neuronal differentiation, suggesting that *HUWE1* abnormalities may affect brain development through cellular stress and differentiation defects [35]. *HUWE1*-mediated degradation of TTBK2 further links this ligase to primary cilium disassembly and cerebellar granule neuron progenitor differentiation [36].

**Figure 3:**
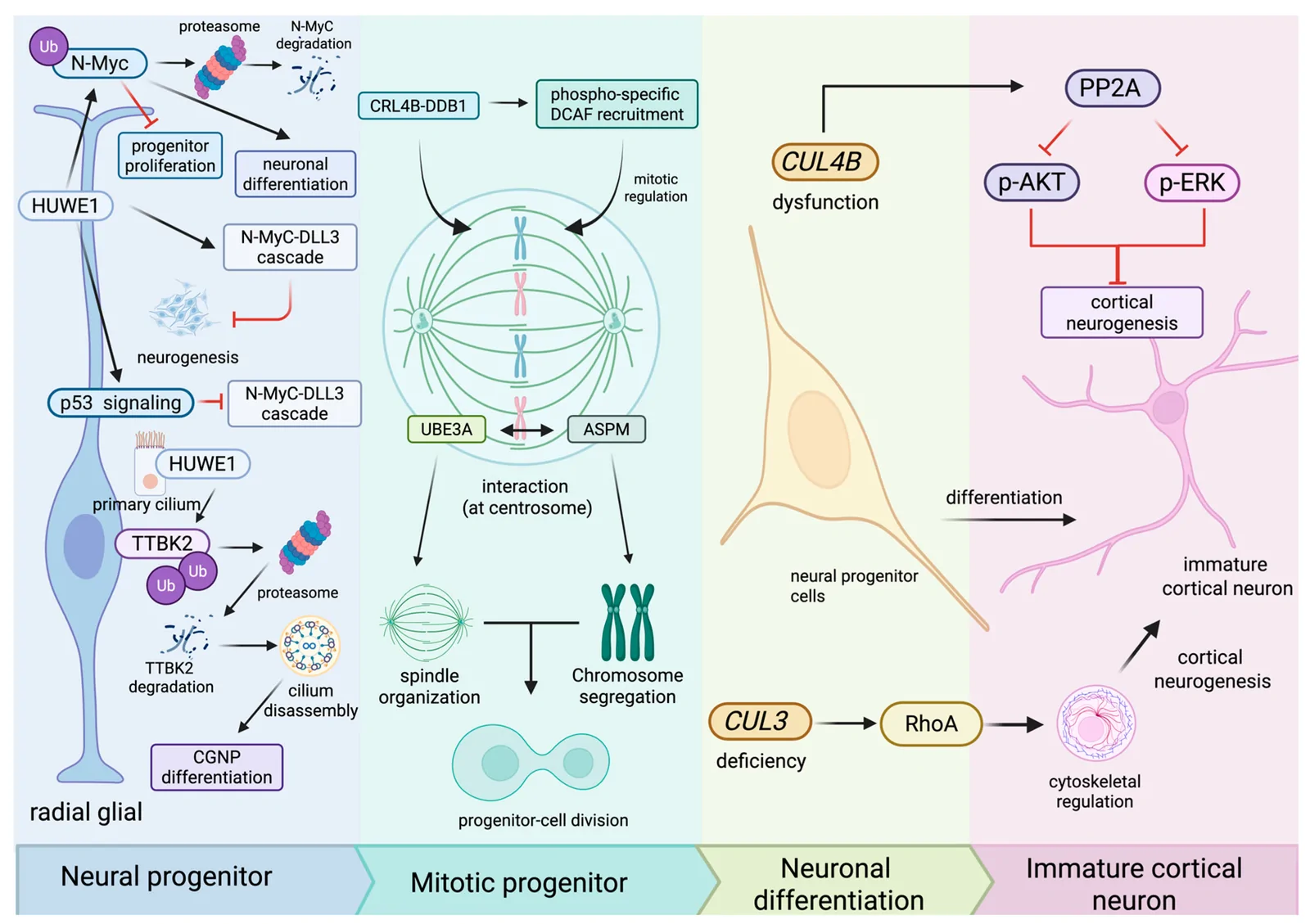
E3 ubiquitin-related regulation of early neurogenesis and progenitor-cell development.

*UBE3A* interacts with ASPM and localizes to centrosomes, linking *UBE3A* to spindle organization and chromosome segregation in progenitor-cell division [84]. *CUL3* affects cytoskeletal dynamics and cortical neurogenesis through RhoA signaling, and *Cul3* haploinsufficiency can alter RhoA-mediated cytoskeletal regulation [42]. These findings broaden E3-related NDD mechanisms beyond mature synapses to include cell number, cell fate, and early brain-structure formation (Figure 3).

### 3.3. Neuronal Migration, Cytoskeletal Regulation, and Cortical Organization

Neuronal migration and cortical organization require coordinated cytoskeletal remodeling, cell polarity, and developmental signaling, allowing newly generated neurons to reach appropriate positions within the developing cortex [85]. Among E3-related NDD genes, *NEDD4L* has a relatively direct connection with this process. Pathogenic *NEDD4L* variants have been linked to periventricular nodular heterotopia and related developmental phenotypes [65], with subsequent clinical observations further expanding the associated phenotypic spectrum [66]. In the developing cortex, abnormal *NEDD4L* activity is associated with altered AKT-mTOR signaling, abnormal neuronal positioning, and impaired terminal translocation [65]. Thus, *NEDD4L* provides a relatively clear example of how E3 dysfunction can be linked to abnormal neuronal positioning and cortical-structure formation.

A more concentrated line of evidence comes from the *CUL3*-CRL3 adaptor system. As a CRL3 scaffold, *CUL3* relies on BTB-domain adaptors for substrate recognition. Loss-of-function variants affecting *CUL3* itself, or missense variants that disrupt *CUL3*-BTB adaptor interaction, provide a complex-level explanation for downstream abnormalities in cytoskeleton-related signaling [38,40]. Cul3 haploinsufficiency can alter cytoskeletal dynamics and cortical neurogenesis through RhoA signaling [42,43]. During critical periods of brain development, Cul3 also regulates cytoskeletal protein homeostasis and cell migration [43]. Bacurd/Kctd13-related adaptors are linked to Rnd/Rho GTPase-related regulation and influence neuronal positioning and dendritic maturation [86]. The Bacurd2-Rnd2 axis is further involved in the radial migration of developing cortical neurons [87]. In a 16p11.2 protein-network analysis, *KCTD13*, *CUL3*, and RhoA were also placed within a shared context of mid-fetal cortical development and psychiatric-disease-related biology [88]. Together, these findings make *CUL3*-related mechanisms an important link between E3-complex dysfunction, Rho-related cytoskeletal regulation, and abnormal neuronal migration.

**Figure 4:**
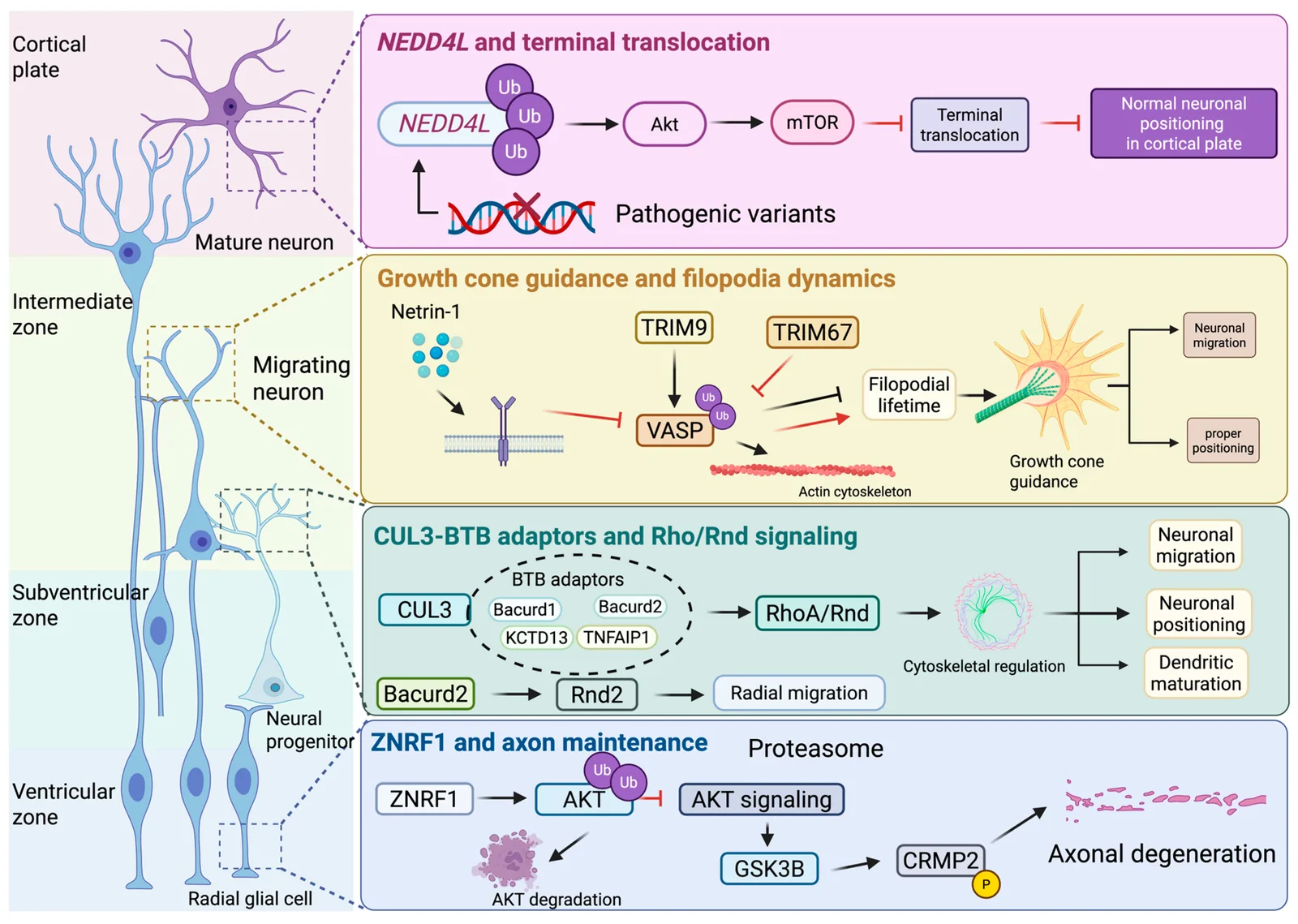
E3 ubiquitin-related pathways in neuronal migration, cytoskeletal regulation, and cortical organization.

Axon guidance and neurite morphology further show that the influence of ubiquitination on cytoskeletal dynamics is not limited to migration [85]. TRIM9 binds to the netrin receptor DCC and ubiquitinates VASP. This modification does not directly promote VASP degradation but instead changes its localization and stability in growth-cone filopodia, thereby restricting filopodial lifetime and participating in netrin-dependent axon guidance [89]. After netrin stimulation, VASP ubiquitination decreases and filopodial stability increases. TRIM67 can be recruited to filopodial tips and prolong filopodial lifetime by antagonizing VASP ubiquitination [90]. ZNRF1 promotes AKT degradation and enhances GSK3B-dependent CRMP2 phosphorylation; inhibition of this pathway delays axon degeneration, indicating that the ZNRF1-AKT-GSK3B-CRMP2 axis contributes to the maintenance of axonal integrity and to axonal degeneration after injury [91]. These mechanistic clues place neuronal migration, cytoskeletal dynamics, and axonal processes within the same developmental trajectory and further suggest that E3-related pathways can influence cortical organization and neural-circuit formation through distinct molecular routes (Figure 4).

### 3.4. Synaptic Development, E/I Balance, and Neural Circuit Maturation

Synaptic development and neural-circuit maturation provide a relatively direct level at which E3 dysfunction can be linked to NDD-related phenotypes [92]. Unlike neuronal positioning and cortical-structure formation, this stage is no longer concerned only with whether neurons reach the correct location, but also with whether connected neurons can maintain appropriate synaptic strength, excitability, and network stability [92]. *UBE3A* is the gene with the most concentrated evidence in this area. Maternal *UBE3A* loss in AS can disrupt multiple aspects of synaptic regulation, among which ion-channel homeostasis is a relatively well-defined mechanism. Loss of *UBE3A* increases synaptic SK2 channel levels, impairs NMDAR-dependent synaptic plasticity, and is associated with learning and memory deficits [93,94]. Regulation of Kv4.2 reflects an activity-dependent mechanism: neuronal activity enhances the interaction between *UBE3A* and Kv4.2 and promotes a reduction in Kv4.2 protein levels, whereas this degradation process is weakened in AS models, affecting the regulation of neuronal excitability and synaptic plasticity [95]. The influence of *UBE3A* on synaptic function is not limited to ion-channel degradation. Mono-ubiquitination of Rabphilin 3A provides an example of non-degradative ubiquitination affecting synaptic function [96]. In human cortical neurons, *UBE3A* loss increases proteins such as GRIPAP1 and PACSIN1, which are involved in AMPA receptor endocytic recycling, thereby linking synaptic protein turnover with activity-dependent synaptic plasticity [97]. Developmental synaptic remodeling is also sensitive to *UBE3A* dosage. Presynaptic *UBE3A* promotes synapse elimination by downregulating BMP signaling, and either insufficient or excessive *UBE3A* activity can alter the timing and extent of synapse elimination [98]. These findings indicate that *UBE3A* dysfunction does not simply alter a single synaptic substrate but can simultaneously affect ion-channel homeostasis, receptor recycling, and developmental synaptic pruning.

Extending beyond *UBE3A* to E3-complex dysfunction, evidence from *CUL3* and *CUL4B* more strongly highlights consequences at the level of excitatory/inhibitory balance and neural networks [99]. *Cul3* deficiency enhances glutamatergic transmission and neuronal excitability and alters E/I balance through cap-dependent translation. Inhibition of the eIF4E-eIF4G1 complex or reduction of neuronal activity partially improves social deficits, spine density, and E/I imbalance-related phenotypes [100]. Region-specific *Cul3* deficiency further shows that loss of the same E3 scaffold can produce different consequences across brain regions: prefrontal cortex alterations are more closely related to social deficits, whereas striatal-circuit alterations are more closely associated with stereotypic behaviors [101]. Evidence related to *CUL4B* places E3-complex abnormalities more specifically in synaptic structure and inhibitory transmission. Nervous system-specific *Cul4b* deficiency causes hippocampal synapse loss, dendritic spine abnormalities, and reduced AMPAR-mediated EPSCs, accompanied by impaired spatial learning and memory [48]. A more specific mechanism involves GABA transporter 1: *Cul4b* deletion leads to GAT1 protein accumulation, enhances GABA reuptake, and thereby weakens GABA-mediated inhibitory synaptic transmission; in the same model, the GAT1 inhibitor tiagabine reduced seizure susceptibility and spontaneous seizures [49]. These findings make the connection between E3-complex dysfunction, synaptic excitability imbalance, impaired inhibitory transmission, and epilepsy-related phenotypes more specific.

**Figure 5:**
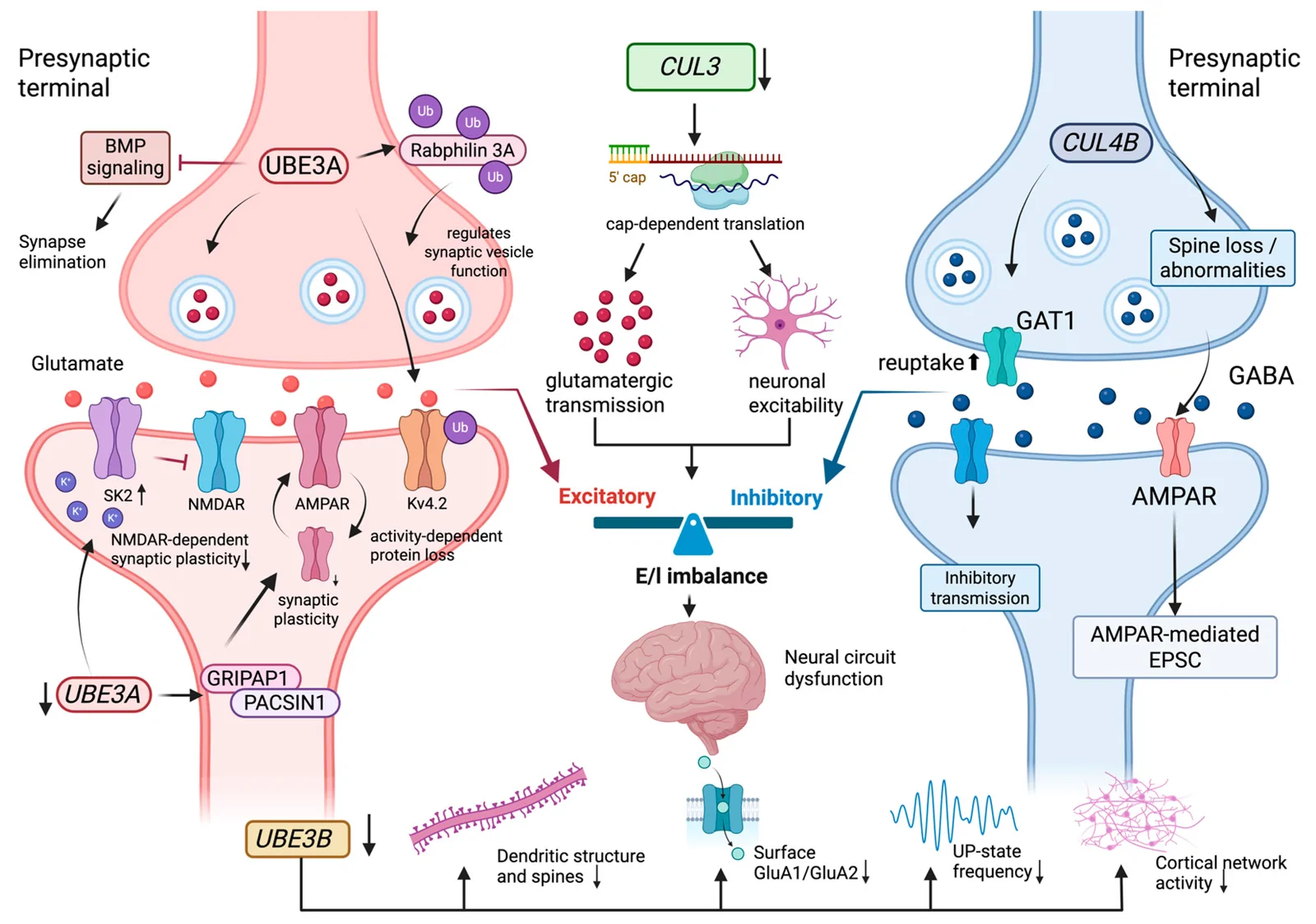
E3 ubiquitin-related mechanisms in synaptic development and neural circuit maturation.Arrows indicate regulatory direction or downstream effects; blunt-ended lines indicate inhibition; upward and downward arrows indicate increased and decreased levels or activity, respectively.

*UBE3B* provides another line of evidence linking HECT-type E3 ligases to synaptic development and cortical network maturation [61]. After CNS-specific *Ube3b* loss, cortical pyramidal neurons show reduced dendritic complexity, dendritic length, spine density, and excitatory synapse density. *Ube3b*-deficient cortices also show reduced UP-state frequency and decreased GluA1/GluA2 surface expression, suggesting that *Ube3b* loss can weaken excitatory synaptic function and cortical circuit activity [102]. Although these mechanisms are not identical, they point to the same broader issue: E3-related abnormalities can alter the formation, pruning, and mature homeostatic maintenance of synaptic connections, making neural circuits more vulnerable to excitability imbalance, cognitive-behavioral abnormalities, or seizure susceptibility (Figure 5).

### 3.5. Proteostasis, Metabolic Regulation, and Cellular Homeostasis

E3 abnormalities can also reshape broader proteostasis and metabolic states. Cross-species, brain-region, and developmental-stage proteomics in *UBE3A*-deficient models indicate dynamic molecular changes: early alterations involve proteasome pathways, translation-related processes, and aminoacyl-tRNA synthetase pathways, whereas synapse-related protein changes become more prominent in adulthood. Some proteomic abnormalities can improve after *UBE3A* restoration [103]. Metabolomic studies similarly show bioenergetic abnormalities in AS mouse embryos, including changes in glycolysis, pyruvate metabolism, the TCA cycle, and mitochondrial function [104]. *UBE3A*-related mechanisms further include Golgi acidification and sialylation [105], PEG10 homeostasis and UBQLN2-dependent proteasomal degradation of PEG10 [106,107], extracellular vesicle dysfunction associated with synaptic and cognitive deficits in AS models [108], extracellular *UBE3A* [109], and autophagy linked to AMPK-ULK1 and p53 signaling [110]. AS pathophysiology should therefore be considered across synaptic, proteostatic, metabolic, and trafficking dimensions.

**Figure 6:**
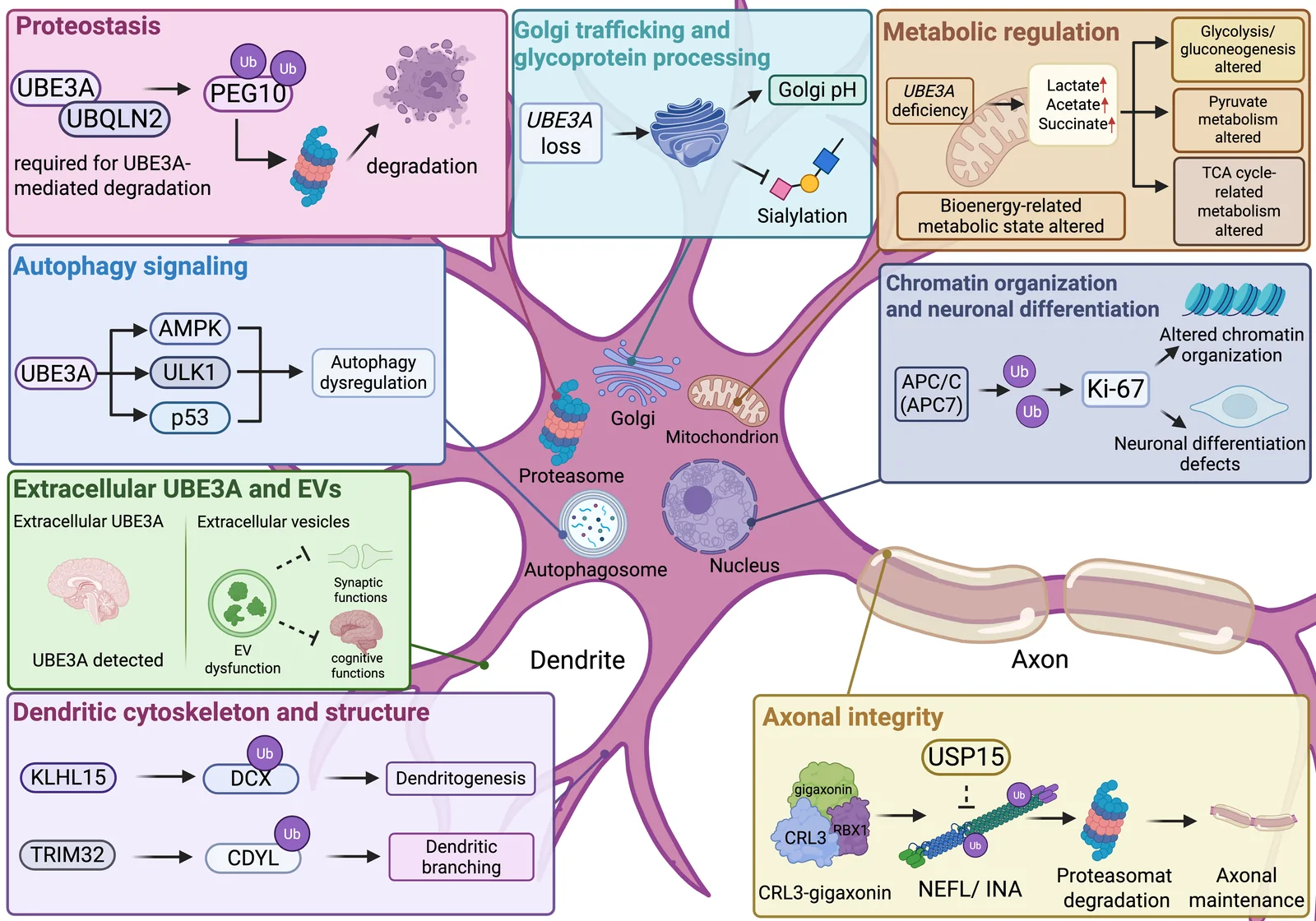
E3 ubiquitin-related regulation of proteostasis, metabolism, and cellular homeostasis.

Other E3-related mechanisms link protein homeostasis, metabolism, chromatin state, and neuronal structure. The CRL3 adaptor *KCTD13* regulates ADSS ubiquitination and purine metabolism, connecting 16p11.2-associated ASD risk with metabolic homeostasis [111]. APC7 deficiency impairs ubiquitin-dependent clearance of chromatin-associated proteins such as Ki-67, thereby placing protein turnover at the interface of cell-cycle exit, chromatin organization, and neuronal differentiation [112]. KLHL15 restricts dendritogenesis by degrading doublecortin [113], TRIM32 promotes dendritic branching by degrading CDYL [114], and the CRL3-gigaxonin-USP15 pathway regulates neurofilament clearance and axonal cytoskeletal maintenance [115]. These examples reinforce the fact that E3-related mechanisms should not be reduced to generalized protein degradation. However, changes in protein abundance, ubiquitination, or metabolites do not automatically establish a direct substrate relationship. Interaction, ubiquitination-site, stability, structural, and functional rescue experiments are needed to distinguish direct substrates from secondary downstream alterations (Figure 6).

**Table 1:** Genetic evidence linking E3 ubiquitin ligases and ubiquitination-related components to neurodevelopmental disorders.

Gene	E3-Related Category	Associated Disorder/Syndrome	Variant or Genetic Mechanism	Inheritance Pattern	Main NDD-Related Features	Genetic Evidence Summarized	References
*UBE3A*	HECT-type E3 ligase	Angelman syndrome	Maternal 15q11-q13 deletion, *UBE3A* variant, paternal UPD, imprinting defect	Maternal allele/imprinting	DD, ID, limited speech, ataxia, seizures, sleep and behavioral features	Clinical genetics and review synthesis	[17]
*UBE3A*	HECT-type E3 ligase	Angelman syndrome	Clinical diagnosis of *UBE3A*-related/imprinting disorder	Usually maternal allele or imprinting defect	Atypical early presentation of AS	Human case report	[18]
*UBE3A*	HECT-type E3 ligase	Angelman syndrome	AS-related genetic etiologies	Maternal allele/imprinting	Developmental milestones and daily living impairment across age	Human cohort/clinical assessment	[19]
*UBE3A*	HECT-type E3 ligase	Angelman syndrome	AS-related genetic etiologies	Maternal allele/imprinting	Autistic traits in the context of AS	Human behavioral cohort	[20]
*UBE3A*	HECT-type E3 ligase	Angelman syndrome	AS-related genetic etiologies	Maternal allele/imprinting	Sleep disturbance and challenging behavior	Human sleep/behavior cohort	[21]
*UBE3A*	HECT-type E3 ligase	*UBE3A*-related ID/DD	Familial pathogenic *UBE3A* variant	Familial inheritance; allele effect requires interpretation	Multigenerational ID and DD	Human family study	[22]
*UBE3A*	HECT-type E3 ligase	Angelman syndrome mechanistic basis	Paternal *UBE3A* silencing in neurons	Allele-specific expression	Basis for neuronal *UBE3A* deficiency	Patient-derived neurons	[23]
*UBE3A*	HECT-type E3 ligase	Angelman syndrome imprinting context	Allele-specific Ube3a expression during development	Maternal/paternal allele regulation	Developmental dependence on maternal expression	Mouse brain expression mapping	[24]
*UBE3A*	HECT-type E3 ligase	*UBE3A* imprinting/dosage context	Brain-region and cell-type distribution of *UBE3A*/E6AP and antisense transcript	Allele-specific regulatory background	Developmental brain expression context	Rhesus monkey brain mapping	[25]
*UBE3A*	HECT-type E3 ligase	15q11-q13 duplication syndrome/Dup15q syndrome	Chromosome 15q11.2-q13.1 duplication; increased *UBE3A* dosage context	Usually maternal duplication in classic Dup15q	ASD-related features, DD/ID, abnormal sleep EEG	Human sleep EEG cohort	[26]
*UBE3A*	HECT-type E3 ligase	*UBE3A* gain-of-function-related ASD/ID	*UBE3A* gain-of-function variant	Dominant/dosage-related	ASD and ID	Human genetic report with functional interpretation	[28]
*UBE3A*	HECT-type E3 ligase	*UBE3A* gain-of-function ASD model	Autism-linked gain-of-function mutation	Maternal or paternal inheritance in model context	Behavioral phenotypes and interneuron abnormalities	Mouse model	[29]
*UBE3A*	HECT-type E3 ligase	15q11-q13 duplication syndrome/Dup15q syndrome model	15q11.2-q13.1 duplication model	Dosage-related model	Sleep EEG signatures	Mouse model sleep EEG	[27]
*HUWE1*	HECT-type E3 ligase	*HUWE1*-related X-linked intellectual disability/NDD	Whole-gene duplication or copy-number gain involving *HUWE1*	X-linked dosage alteration	ID and syndromic developmental phenotype	Human CNV/family genetics	[31]
*HUWE1*	HECT-type E3 ligase	*HUWE1*-related NDD/X-linked intellectual disability context	Pathogenic *HUWE1* sequence variants	X-linked	DD, ID, speech impairment, seizures, autistic features, hypotonia	Human cohort genetics	[32]
*HUWE1*	HECT-type E3 ligase	*HUWE1*-related NDD/X-linked intellectual disability context	Pathogenic *HUWE1* variants	X-linked	DD, ID, speech/language impairment and variable syndromic features	Human cohort and suggested evaluations	[33]
*CUL3*	Cullin-RING ligase scaffold	Neurodevelopmental disorder with or without autism or seizures	Heterozygous/de novo *CUL3* variants	Autosomal dominant/de novo	DD, ID, ASD-related features, seizures	Human cohort genetics	[37]
*CUL3*	Cullin-RING ligase scaffold	*CUL3*-related disorder / NEDAUS spectrum	Missense variants affecting BTB-adaptor binding	Autosomal dominant/de novo	Diverse NDD phenotypes	Human genetics plus binding assay	[40]
*CUL3*	Cullin-RING ligase scaffold	*CUL3*-related syndromic NDD/NEDAUS	Loss-of-function variants	Autosomal dominant/de novo	DD/ID and syndromic features	Human cohort genetics	[38]
*CUL3*	Cullin-RING ligase scaffold	*CUL3*-related NDD/NEDAUS	*CUL3* pathogenic variants	Autosomal dominant/de novo	DD, ID, ASD-related features, seizures and variable congenital features	Human cohort and episignature analysis	[39]
*CUL4B*	Cullin-RING ligase scaffold/core component	X-linked intellectual developmental disorder, Cabezas type	Pathogenic *CUL4B* variants	X-linked	ID, speech/motor delay, seizures and syndromic features	Human genetics	[44]
*CUL4B*	Cullin-RING ligase scaffold/core component	*CUL4B*-associated XLID/Cabezas spectrum	Pathogenic *CUL4B* variants	X-linked	ID, brain malformations and syndromic features	Human cohort genetics	[45]
*HECW2*	HECT-type E3 ligase	*HECW2*-related NDD	De novo missense variants	Autosomal dominant/de novo	Neurodevelopmental delay, ID, hypotonia	Human genetics with functional context	[50]
*HECW2*	HECT-type E3 ligase	Developmental and epileptic encephalopathy in *HECW2*-related reports	*HECW2* variants; zebrafish hecw2a knockdown	Autosomal dominant/de novo; model knockdown	Epilepsy, DD and abnormal brain development	Human genetics plus zebrafish model	[51]
*HECW2*	HECT-type E3 ligase	*HECW2*-related developmental delay/NDD	Novel *HECW2* variant	Likely de novo/dominant in reported case context	DD, neurodevelopmental delay, hypotonia	Human case report	[52]
*TRIP12*	HECT-type E3 ligase	Clark-Baraitser syndrome/TRIP12-related NDD	TRIP12 pathogenic variants/haploinsufficiency	Autosomal dominant/de novo	DD, ID, speech delay, behavioral abnormalities	Human cohort genetics	[54]
*TRIP12*	HECT-type E3 ligase	Clark-Baraitser syndrome/TRIP12-related NDD	TRIP12 haploinsufficiency or pathogenic variants	Autosomal dominant/de novo	ID, DD, speech impairment and ASD-related features	Human genetics	[55]
*TRIP12*	HECT-type E3 ligase	TRIP12-related NDD/Clark-Baraitser syndrome	TRIP12 variants	Autosomal dominant/de novo	DD, ID, speech impairment, behavioral problems and facial phenotype	Human cohort phenotype study	[56]
*UBE3B*	HECT-type E3 ligase	Kaufman oculocerebrofacial syndrome	*UBE3B* deficiency/biallelic loss-of-function	Autosomal recessive	Severe ID, DD, ocular/facial anomalies, congenital features	Human genetics plus model organism data	[57]
*UBE3B*	HECT-type E3 ligase	Blepharophimosis-ptosis-intellectual-disability syndrome/KOS	Biallelic *UBE3B* variants	Autosomal recessive	ID, growth delay and multisystem congenital features	Human genetics	[58]
*UBE3B*	HECT-type E3 ligase	Kaufman oculocerebrofacial syndrome/*UBE3B*-related syndrome	Biallelic *UBE3B* pathogenic variants	Autosomal recessive	DD/ID, microcephaly, hypotonia and syndromic features	Human case series	[59]
*UBE3B*	HECT-type E3 ligase	Kaufman oculocerebrofacial syndrome/*UBE3B*-related syndrome	Novel biallelic *UBE3B* mutations	Autosomal recessive	DD/ID and additional dysmorphic/congenital findings	Human case series	[60]
*UBR5*	N-recognin/HECT-type E3 ligase	*UBR5*-related neurodevelopmental syndrome	Heterozygous pathogenic *UBR5* variants	Autosomal dominant/often de novo	DD, ASD, ID, speech/language impairment and epilepsy	Human cohort genetics	[62]
*UBR5*	N-recognin/HECT-type E3 ligase	ASD/ID associated with *UBR5* loss-of-function	*UBR5* loss-of-function variants	Autosomal dominant/de novo or inherited context	ASD and ID with variable NDD features	Human case series and literature review	[63]
*UBR5/UBR7*	N-recognin-related E3 factors	UBR7/*UBR5*-associated Notch-related neurodevelopmental syndrome	UBR7 disease gene with *UBR5* participation in Notch pathway	Mostly de novo/dominant for UBR7 syndrome context	Epilepsy, ptosis, hypothyroidism and DD/ID-related features	Human genetics plus functional assays	[64]
*NEDD4L*	HECT-type E3 ligase	Periventricular nodular heterotopia 7	Pathogenic *NEDD4L* variants	Autosomal dominant/de novo or inherited in reported families	DD/ID, epilepsy, cortical malformation, speech/motor abnormalities	Human genetics plus functional/model data	[65]
*NEDD4L*	HECT-type E3 ligase	*NEDD4L*-related disorder/PVNH7	Pathogenic *NEDD4L* variant	Autosomal dominant/reported case context	PVNH, DD, syndromic features and ophthalmic finding	Human case report and literature summary	[66]
*RHOBTB2*	BTB-domain atypical Rho GTPase; *CUL3*-related ubiquitination context	Developmental and epileptic encephalopathy 64	Pathogenic *RHOBTB2* variants	Autosomal dominant/de novo	Early-onset epilepsy, DD/ID, movement disorder, hypotonia/dystonia	Human genetics	[67]
*RHOBTB2*	BTB-domain atypical Rho GTPase; *CUL3*-related ubiquitination context	*RHOBTB2*-associated NDD/DEE64 spectrum	Pathogenic *RHOBTB2* variants at different protein positions	Autosomal dominant/mostly de novo	Epilepsy, DD/ID, motor and tone abnormalities	Human genotype–phenotype cohort	[68]
*FBXO11*	F-box substrate receptor in SCF-type CRL complex	*FBXO11*-related IDDFBA/NDD	De novo missense variants altering protein behavior	Autosomal dominant/de novo	ID/DD and variable syndromic features	Human genetics plus in vitro functional assays	[70]
*FBXW7*	F-box substrate receptor in SCF-type CRL complex	*FBXW7* neurodevelopmental syndrome	Heterozygous pathogenic *FBXW7* variants	Autosomal dominant/de novo	Global DD, ID, hypotonia, speech delay, epilepsy and brain anomalies	Human genetics plus functional assays	[71]
*FBXW7*	F-box substrate receptor in SCF-type CRL complex	*FBXW7*-related NDD/*FBXW7* neurodevelopmental syndrome	Pathogenic *FBXW7* variants	Autosomal dominant/de novo or inherited context	DD/ID, hypotonia, language delay and variable congenital features	Human case report/series	[72]
*RNF2/RING1*	Polycomb-associated RING-type E3 ligase/PRC1 component	*RNF2*-related syndromic neurodevelopmental disorder	De novo *RNF2* variants	Autosomal dominant/de novo	DD/ID, behavioral abnormalities, seizures and congenital/syndromic features	Human genetics and chromatin-regulation context	[74]
*RING1*	Polycomb-associated RING-type E3 ligase/PRC1 component	*RING1*-related neurodevelopmental/chromatin disorder	Missense variants affecting *RING1* function	Autosomal dominant/de novo	Neurodevelopmental abnormalities with syndromic features	Human variants plus functional neurogenesis assays	[75]
*RNF2/RING1*	Polycomb-associated RING-type E3 ligase/PRC1 component	Polycomb-related NDD involving *RNF2*/*RING1*	Variants altering chromatin binding of Polycomb complexes	Autosomal dominant/de novo context	Syndromic neurodevelopmental disorders	Human genetics plus chromatin/model studies	[76]
*IRF2BPL*	Transcriptional/ubiquitination-associated regulator	Neurodevelopmental disorder with regression, abnormal movements, loss of speech and seizures	Pathogenic/truncating *IRF2BPL* variants	Autosomal dominant/de novo	DD/ID, regression, movement disorder, speech loss, seizures	Human genotype–phenotype study	[77]
*IRF2BPL*	Transcriptional/ubiquitination-associated regulator	*IRF2BPL* syndrome/NEDAMSS	Pathogenic *IRF2BPL* variants	Autosomal dominant/de novo or inherited context	DD/ID, regression, epilepsy, ataxia and abnormal movements	Human cohort genetics	[78]
*KCTD13*	BTB/KCTD adaptor linked to *CUL3*-type ubiquitination	16p11.2 CNV-associated neurodevelopmental phenotype	16p11.2 copy-number alteration involving KCTD13; KCTD13 dosage	CNV dosage-related	ASD-related features and developmental/metabolic phenotype	Human CNV/proteomic or cellular model context	[111]
*KCTD13*	BTB/KCTD adaptor linked to *CUL3*-type ubiquitination	16p11.2 CNV-associated neurodevelopmental phenotype	16p11.2 CNV network involving KCTD13	CNV dosage-related	Psychiatric/NDD risk with cortical developmental context	Spatiotemporal protein network analysis	[88]
*KLHL15*	Kelch-like substrate adaptor / E3 ligase-related protein	KLHL15-related X-linked intellectual disability	KLHL15 pathogenic variant	X-linked	Intellectual disability and neurodevelopmental impairment	Human disease context plus biochemical/cell assays	[113]

**Table 2:** Mechanistic roles of E3 ubiquitin ligases and ubiquitination-related components in neurodevelopmental and cellular processes.

Process Category	E3-Related Gene	Molecular Module/Key Substrate or Pathway	Main Functional Findings	Neurodevelopmental Relevance	Evidence Model or System	References
Neural progenitor proliferation, differentiation, and early brain development	*CUL4B*	NPC growth and cell-cycle regulation	*CUL4B* regulates neural progenitor growth and cell-cycle control.	Supports early progenitor expansion and cortical neurogenesis.	Neural progenitor cell models	[81]
Neural progenitor proliferation, differentiation, and early brain development	*CUL4B*	PP2A-dependent AKT/ERK signaling	*CUL4B* mutations impair cortical neurogenesis through PP2A-dependent inhibition of AKT and ERK signaling.	Links *CUL4B* dysfunction to impaired human cortical neurogenesis.	Human cortical neurogenesis models	[47]
Neural progenitor proliferation, differentiation, and early brain development	CRL4B-DDB1 complex	Phosphorylation-specific DCAF recruitment	*CUL4B*-based E3 complexes recruit phosphorylation-specific DCAFs during mitotic regulation.	Connects CRL4B complex function with mitosis and brain development.	Mitotic and developmental model systems	[82]
Neural progenitor proliferation, differentiation, and early brain development	*HUWE1*	N-Myc degradation	*HUWE1* controls N-Myc stability and thereby regulates neural progenitor proliferation and neuronal differentiation.	Places HECT-type E3 activity in early cell-number and differentiation control.	Developing brain and neural differentiation models	[34]
Neural progenitor proliferation, differentiation, and early brain development	*HUWE1*	N-Myc-DLL3 cascade	*HUWE1* suppresses the N-Myc-DLL3 cascade to inhibit proliferation and promote neurogenesis.	Links *HUWE1*-dependent N-Myc regulation to neurogenic progression.	Brain tumor and neurogenesis models	[83]
Neural progenitor proliferation, differentiation, and early brain development	*HUWE1*	p53 signaling	Enhanced p53 signaling impairs neuronal differentiation in *HUWE1*-associated intellectual disability models.	Connects *HUWE1* dysfunction with differentiation defects in a disease-relevant context.	Models of *HUWE1*-associated intellectual disability	[35]
Neural progenitor proliferation, differentiation, and early brain development	*HUWE1*	TTBK2 and primary cilium disassembly	*HUWE1*-mediated TTBK2 degradation regulates primary cilium disassembly and cerebellar granule neuron progenitor differentiation.	Links protein turnover to ciliary dynamics and cerebellar development.	Cerebellar development and progenitor models	[36]
Neural progenitor proliferation, differentiation, and early brain development	*UBE3A*	ASPM interaction and centrosome localization	*UBE3A* interacts with ASPM and localizes to centrosomes, with links to spindle organization and chromosome segregation.	Suggests a role for *UBE3A* in progenitor-cell division-related processes.	Cell-based localization and interaction assays	[84]
Neural progenitor proliferation, differentiation, and early brain development	*CUL3*	RhoA signaling and cytoskeletal dynamics	Cul3 haploinsufficiency alters RhoA-related cytoskeletal regulation and cortical neurogenesis.	Connects CRL3 dysfunction with early cortical development and cytoskeletal control.	Mouse and neuronal model systems	[42]
Neuronal migration, cytoskeletal regulation, and cortical organization	*NEDD4L*	AKT-mTOR signaling and terminal translocation	*NEDD4L* activity is linked to AKT-mTOR signaling, neuronal positioning, and terminal translocation in the developing cortex.	Provides a mechanism-oriented link to cortical malformation and abnormal neuronal positioning.	Developing cortex and patient-genetic studies	[65,66]
Neuronal migration, cytoskeletal regulation, and cortical organization	*CUL3 / CRL3* adaptor system	BTB-domain adaptor interaction	*CUL3* loss-of-function variants and variants affecting *CUL3*-BTB adaptor interaction provide a complex-level basis for downstream cytoskeletal effects.	Connects scaffold or adaptor-binding defects with cytoskeletal pathway disruption.	Patient-genetic and biochemical studies	[38,40]
Neuronal migration, cytoskeletal regulation, and cortical organization	*CUL3*	Cytoskeletal protein homeostasis and cell migration	Cul3 regulates cytoskeletal protein homeostasis and cell migration during a critical developmental window.	Links CRL3 function to neuronal migration and cortical organization.	Developing brain and cell migration models	[43]
Neuronal migration, cytoskeletal regulation, and cortical organization	Bacurd/*Kctd13*-related adaptors	Rnd/Rho GTPase-related regulation	Bacurd1/Kctd13 and Bacurd2/Tnfaip1 interact with Rnd proteins and influence neuronal positioning and dendritic maturation.	Places CRL3 adaptors in Rho-family cytoskeletal regulation during cortical development.	Cortical neuron developmental models	[86]
Neuronal migration, cytoskeletal regulation, and cortical organization	*Bacurd2*	Rnd2-dependent radial migration	Bacurd2 interacts with Rnd2 and controls radial migration in the developing mammalian cortex.	Links adaptor-mediated Rnd signaling to neuronal migration.	Developing mammalian cerebral cortex	[87]
Neuronal migration, cytoskeletal regulation, and cortical organization	*KCTD13-CUL3*-RhoA pathway	16p11.2 protein network	KCTD13, *CUL3*, and RhoA are connected in a spatiotemporal protein network involving mid-fetal cortical development.	Provides network-level support for RhoA-related cortical developmental biology.	Spatiotemporal protein-network analysis	[88]
Neuronal migration, cytoskeletal regulation, and cortical organization	*TRIM9/TRIM67*	DCC, VASP ubiquitination, and filopodial stability	TRIM9 ubiquitinates VASP in a non-degradative manner, whereas TRIM67 counteracts VASP ubiquitination to regulate filopodial lifetime.	Links ubiquitination to growth-cone dynamics and netrin-dependent axon guidance.	Neuronal growth-cone and axon-guidance assays	[89,90]
Neuronal migration, cytoskeletal regulation, and cortical organization	*ZNRF1*	AKT-GSK3B-CRMP2 axis	ZNRF1 promotes AKT degradation, thereby enhancing GSK3B-dependent CRMP2 phosphorylation during axon degeneration.	Connects ubiquitin-mediated protein turnover with axonal integrity.	Axon injury and degeneration models	[91]
Synaptic development, E/I balance, and neural circuit maturation	*UBE3A*	SK2 channel regulation	*UBE3A* loss increases synaptic SK2 channel levels and impairs NMDAR-dependent synaptic plasticity.	Links *UBE3A* deficiency with altered synaptic plasticity and learning-related circuitry.	Angelman syndrome mouse and hippocampal models	[93,94]
Synaptic development, E/I balance, and neural circuit maturation	*UBE3A*	Kv4.2 activity-dependent degradation	*UBE3A* binds and ubiquitinates Kv4.2, promoting activity-dependent Kv4.2 protein loss.	Connects *UBE3A*-dependent protein turnover with neuronal excitability and synaptic plasticity.	Angelman syndrome mouse and neuronal activity models	[95]
Synaptic development, E/I balance, and neural circuit maturation	*UBE3A*	Rabphilin 3A monoubiquitination	*UBE3A* mediates non-degradative monoubiquitination of Rabphilin 3A.	Provides a non-degradative ubiquitination mechanism relevant to presynaptic function.	Biochemical and synaptic-function assays	[96]
Synaptic development, E/I balance, and neural circuit maturation	*UBE3A*	GRIPAP1, PACSIN1, and AMPA receptor recycling	*UBE3A* regulates GRIPAP1 and PACSIN1 proteins linked to AMPA receptor endocytic recycling.	Connects *UBE3A* loss with receptor trafficking and synaptic plasticity.	Human cortical neuron and cell-based assays	[97]
Synaptic development, E/I balance, and neural circuit maturation	*UBE3A*	BMP signaling and synapse elimination	Presynaptic Ube3a promotes synapse elimination through downregulation of BMP signaling.	Links E3 activity with developmental synapse remodeling.	Synapse-development model systems	[98]
Synaptic development, E/I balance, and neural circuit maturation	*CUL3*	Cap-dependent translation and eIF4E-eIF4G1 complex	*CUL3* deficiency enhances cap-dependent translation; modulation of eIF4E-eIF4G1 or neuronal activity improves selected synaptic and behavioral readouts.	Connects CRL3 dysfunction with glutamatergic transmission, neuronal excitability, and E/I balance.	Cul3-deficient mouse and neuronal models	[100]
Synaptic development, E/I balance, and neural circuit maturation	*CUL3*	Region-specific circuit effects	Prefrontal and striatal Cul3 deficiency produce partly distinct effects on social and stereotypic behavior-related circuitry.	Links E3-complex dysfunction with brain-region-specific circuit maturation.	Region-specific Cul3 mouse models	[101]
Synaptic development, E/I balance, and neural circuit maturation	*CUL4B*	Synapse loss, spine abnormalities, and AMPAR-mediated EPSCs	Neural Cul4b deficiency causes synapse loss, dendritic spine abnormalities, and reduced AMPAR-mediated EPSCs.	Links *CUL4B* dysfunction with excitatory synaptic function.	Neural-specific Cul4b mouse models	[48]
Synaptic development, E/I balance, and neural circuit maturation	*CUL4B*	GAT1 accumulation and GABA reuptake	Cul4b deletion causes GAT1 accumulation, increased GABA reuptake, and impaired inhibitory synaptic transmission.	Connects CRL4B dysfunction with inhibitory transmission and epilepsy-related network vulnerability.	Cul4b-deficient mice and pharmacological assays	[49]
Synaptic development, E/I balance, and neural circuit maturation	*UBE3B*	Dendritic complexity, spine density, surface GluA1/GluA2, and cortical UP states	CNS-specific Ube3b loss reduces dendritic complexity, excitatory synapse density, surface GluA1/GluA2, and cortical UP-state frequency.	Places *UBE3B* in excitatory synaptic development and cortical network maturation.	CNS-specific Ube3b mouse models	[102]
Proteostasis, metabolic regulation, and cellular homeostasis	*UBE3A*	Spatiotemporal proteomic changes	*UBE3A*-deficient models show developmental and region-dependent proteomic changes involving proteasome, translation-related, aminoacyl-tRNA synthetase, and synapse-related pathways.	Links *UBE3A* loss with proteostasis and synapse-related molecular maturation.	Cross-species, brain-region, and developmental-stage proteomics	[103]
Proteostasis, metabolic regulation, and cellular homeostasis	*UBE3A*	Glycolysis/gluconeogenesis, pyruvate metabolism, TCA cycle-related metabolism, and mitochondrial function-related annotations	Early AS model metabolomics showed altered bioenergy-related metabolic pathways, including increased lactate, acetate, and succinate.	Provides annotation-level evidence for altered metabolic state during early development.	1H-NMR metabolomics in AS mouse embryos	[104]
Proteostasis, metabolic regulation, and cellular homeostasis	*UBE3A*	Golgi acidification and surface protein sialylation	*UBE3A* loss affects Golgi acidification and cell-surface protein sialylation.	Links *UBE3A* dysfunction with trafficking and glycoprotein processing.	Cellular and neuronal assays	[105]
Proteostasis, metabolic regulation, and cellular homeostasis	*UBE3A*/UBQLN2	PEG10 proteasomal degradation	UBQLN2 contributes to *UBE3A*-mediated proteasomal degradation of PEG10.	Connects *UBE3A*-dependent degradation with retroelement-derived protein control.	Proteostasis and cell-based assays	[106,107]
Proteostasis, metabolic regulation, and cellular homeostasis	*UBE3A*	Extracellular vesicles and extracellular *UBE3A*	*UBE3A*-related extracellular vesicle dysfunction and extracellular *UBE3A* detection are linked to synaptic or cognitive functional contexts.	Extends *UBE3A*-related mechanisms to extracellular signaling and trafficking contexts.	AS model extracellular vesicle, CSF, and extracellular-space assays	[108,109]
Proteostasis, metabolic regulation, and cellular homeostasis	*UBE3A*	AMPK-ULK1 and p53-related autophagy signaling	*UBE3A* deficiency is associated with autophagy dysregulation involving AMPK-ULK1 and p53 pathways.	Links *UBE3A* loss with cellular homeostasis pathways.	AS model and cellular assays	[110]
Proteostasis, metabolic regulation, and cellular homeostasis	*KCTD13*/CRL3 adaptor	ADSS ubiquitination and purine metabolism	KCTD13 regulates ADSS ubiquitination and purine metabolism.	Connects a CRL3 adaptor with metabolic homeostasis in 16p11.2-associated ASD biology.	Cellular and metabolic assays	[111]
Proteostasis, metabolic regulation, and cellular homeostasis	APC/C-APC7	Ki-67 and chromatin-associated protein clearance	APC7 supports ubiquitin-dependent clearance of chromatin-associated proteins such as Ki-67.	Links protein clearance with cell-cycle exit, chromatin organization, and neuronal differentiation.	Developing mammalian brain and neural progenitor models	[112]
Proteostasis, metabolic regulation, and cellular homeostasis	*CUL3-KLHL15*	Doublecortin protein degradation	KLHL15 promotes degradation of doublecortin proteins and constrains dendritogenesis.	Links CRL3 substrate-adaptor function to dendritic structure.	Neuronal morphology and biochemical assays	[113]
Proteostasis, metabolic regulation, and cellular homeostasis	*TRIM32*	CDYL degradation	TRIM32 promotes CDYL degradation and dendritic branching.	Connects ubiquitin-mediated degradation with dendritic arborization.	Neuronal morphology assays	[114]
Proteostasis, metabolic regulation, and cellular homeostasis	*CRL3*-gigaxonin/USP15	Neurofilament protein clearance	CRL3-gigaxonin promotes neurofilament clearance, whereas USP15 antagonizes this pathway.	Links E3 complex activity and deubiquitination with axonal cytoskeletal maintenance.	Biochemical and cellular models	[115]

## 4. Functional Evidence and Therapeutic Translation

Functional evidence is essential for determining whether E3-related genetic findings can be interpreted as disease mechanisms or potential therapeutic entry points. For many E3 ligases and E3-complex components, pathogenic variants may affect substrate recognition, ubiquitination, protein stability, complex assembly, or downstream cellular states, but these changes do not carry the same translational weight. Some mechanisms have already produced rescue-sensitive readouts or early therapeutic strategies, whereas others remain candidate substrates or pathway-level observations that require further validation. The following section therefore distinguishes relatively mature therapeutic paradigms from preclinical mechanisms and candidate targets that still need stronger functional support.

### 4.1. UBE3A Restoration and Paternal Reactivation in Angelman Syndrome

Among E3-related NDDs, *UBE3A*-related Angelman syndrome (AS) is currently the direction closest to therapeutic translation. AS is mainly caused by maternal *UBE3A* loss, while paternal *UBE3A* is usually silenced in mature neurons through *UBE3A*-ATS-related antisense transcription [23]. Restoring *UBE3A* expression or reactivating the paternal allele therefore directly targets the disease gene itself [116]. This relatively focused disease mechanism and therapeutic entry point distinguish AS from most other E3-related NDDs. For many E3-related disorders, even after pathogenic variants have been identified, key substrates, affected pathways, and rescue-sensitive phenotypes still need to be defined. In AS, by contrast, the primary problem is insufficient neuronal *UBE3A* expression, allowing therapeutic strategies to focus on how to restore *UBE3A* to an appropriate level.

The most advanced evidence currently comes from antisense oligonucleotide (ASO) strategies targeting *UBE3A-ATS.* ASO-mediated suppression of *UBE3A-ATS* can restore *UBE3A* expression in AS mouse models and improve some neurobehavioral phenotypes [117]. The importance of this finding is not simply that certain model phenotypes can be partially improved but that paternal allele silencing itself can serve as an actionable therapeutic target. Subsequent patient-derived neuron screening and non-human primate studies brought this mechanism closer to human translation and supported the clinical development of rugonersen [118]. A phase 1 trial further advanced the paternal *UBE3A* reactivation strategy into children with AS [119]. Additional ASO-related models have helped address translational issues such as target engagement, delivery, and treatment-response monitoring. A xenotransplantation model can be used to evaluate the target engagement of human-specific ASOs in human AS neurons [120]. A prenatal delivery model addresses brain distribution and the possibility of early intervention [121], while a longitudinal EEG model provides a neurophysiological readout for monitoring treatment response [122]. Thus, ASO-mediated paternal allele reactivation is no longer merely a candidate mechanism but has entered a stage in which preclinical validation and early clinical evaluation are beginning to connect.

This evidence chain still requires cautious interpretation. *UBE3A* restoration in models shows that some molecular and behavioral abnormalities are reversible, but different readouts may require different timing and degrees of restoration. The effects of *UBE3A* reinstatement on behavioral and proteomic abnormalities are timing- and readout-dependent, indicating that “restoring expression” does not automatically mean that all phenotypes can be reversed [123]. This is particularly important for children with AS, because neurodevelopmental abnormalities may leave structural or network-level consequences at different developmental stages. Dosage control and species differences also affect translational interpretation. Insufficient *UBE3A* expression causes AS, whereas increased *UBE3A* dosage is associated with Dup15q and ASD-like phenotypes. The therapeutic goal is therefore not simply to increase *UBE3A* but to restore it to a relatively safe expression range in the appropriate brain regions and developmental windows. Differences in imprinted-region architecture in large-animal models further caution against directly extrapolating regulatory mechanisms that are effective in rodents to humans [25,124]. These issues indicate that, although the ASO approach is currently the most mature, delivery, dosage, developmental timing, and long-term safety still require continued optimization.

Beyond ASOs, AAV-dCas9 and zinc-finger nuclease approaches can also relieve paternal *UBE3A* silencing by blocking *UBE3A-ATS* transcription [125,126]. Topotecan-related mechanisms further suggest that R-loop formation, chromatin state, and antisense transcript architecture influence transcriptional regulation at this imprinted region [127,128]. These approaches broaden the technical routes for paternal *UBE3A* reactivation and also help explain why the *UBE3A-ATS* region can be indirectly targeted by nucleic-acid tools, gene-regulatory tools, or small molecules. Compared with ASOs, these strategies are currently better regarded as emerging preclinical approaches rather than established therapeutic paths. Overall, *UBE3A* reactivation or restoration represents the most mature mechanism-guided therapeutic paradigm among E3-related NDDs because it combines a clear genetic cause, a defined silenced allele, measurable reactivation readouts, and early clinical translational evidence. This paradigm also highlights that therapeutic exploration of developmentally related E3 pathways must carefully address developmental timing, dosage, cell type, and long-term safety.

### 4.2. Preclinical Translational Mechanisms Beyond UBE3A

Unlike *UBE3A*-related AS, most non-*UBE3A* E3-related NDDs currently lack mature strategies for directly restoring disease–gene function. Their therapeutic implications more often depend on whether downstream pathways are modifiable. It is therefore necessary to distinguish general mechanistic descriptions from preclinical mechanisms that already show rescue-related evidence.

*CUL3*-related evidence illustrates this type of “downstream pathway reversibility.” *Cul3* deficiency enhances cap-dependent translation and is accompanied by abnormalities in excitatory transmission, neuronal excitability, and E/I balance. Inhibition of the eIF4E-eIF4G1 complex or reduction of neuronal activity can partially improve social deficits, spine density, and E/I imbalance-related phenotypes [100]. The key point is not only that *Cul3* loss causes synaptic and behavioral abnormalities, but also that translation control and neuronal activity modulation can modify these abnormalities. In other words, *CUL3* itself may not necessarily be a direct drug target, but some downstream network states caused by CRL3 dysfunction may be modifiable. Region-specific *Cul3* deficiency further adds complexity to this mechanism: prefrontal cortex alterations are more closely related to social deficits, whereas striatal-circuit alterations are more closely associated with stereotypic behaviors [101]. This indicates that the translational interpretation of *CUL3*-related mechanisms cannot rely only on whole-brain or systemic rescue but should consider whether brain region, cell type, and behavioral dimension can be matched.

The *CUL4B*-GAT1 axis provides a preclinical example that is closer to pharmacological modulation. *Cul4b* deletion not only affects learning, memory, and synaptic function [48] but also leads to the accumulation of GABA transporter 1 (GAT1), enhances GABA reuptake, and weakens GABA-mediated inhibitory synaptic transmission [49]. This mechanistic chain is more specific than a general description of “synaptic abnormality”: E3-complex dysfunction is linked to transporter dysregulation, transporter dysregulation alters inhibitory transmission, and impaired inhibitory transmission is further connected to seizure susceptibility. In this model, the GAT1 inhibitor tiagabine can reduce seizure susceptibility and spontaneous seizures [49]. The significance of the *CUL4B*-GAT1 axis therefore lies not simply in the identification of a downstream molecule but in the formation of a relatively complete chain from E3 dysfunction to a pharmacologically modifiable phenotype. However, this should still be defined as a preclinical mechanistic clue rather than a mature therapeutic strategy for *CUL4B*-related disorder. Whether it can be generalized across different variant types, developmental stages, or other clinical phenotypes remains to be further validated.

KCTD13-ADSS-purine metabolism is positioned at an earlier stage. As a *CUL3* adaptor-related molecule, KCTD13 can regulate ADSS ubiquitination and connect 16p11.2-associated ASD biology with metabolic homeostasis through purine metabolism [111]. The importance of this finding is that it extends E3-complex adaptor dysfunction beyond traditional issues of neuronal morphology or synaptic function to the regulation of cellular metabolic states. However, compared with the translation/activity rescue evidence for *CUL3* and the pharmacological modulation evidence for *CUL4B*-GAT1, KCTD13-ADSS-purine metabolism currently lacks a clear rescue readout and a directly corresponding therapeutic strategy. It is therefore better placed at the level of a candidate pathway with potential translational relevance, rather than at the same level of maturity as *CUL4B*-GAT1.

### 4.3. Candidate Substrates and Mechanisms Requiring Further Validation

Beyond *UBE3A* reactivation and a small number of pathways with preclinical modulatory evidence, many E3-related mechanisms are currently better viewed as candidate substrates, functional readouts, or targets for further validation. Substrate studies of *UBE3A* provide a typical example. Kv4.2 and rabphilin 3A link *UBE3A*-dependent regulation to neuronal excitability and synaptic function, respectively [95,96], while GRIPAP1 and PACSIN1 further involve AMPA receptor recycling [97]. Other candidate mechanisms more strongly reflect changes in protein homeostasis, such as PEG10 regulation and β-catenin stabilization [106,129]. These findings help dissect different layers of molecular abnormality in AS and may guide the selection of downstream readouts for future experiments, but they do not occupy the same translational position as strategies that directly restore *UBE3A* expression.

A similar situation exists for non-*UBE3A* mechanisms. *UBE3B* loss is associated with synaptic development and cortical network activity [102], whereas mechanisms involving KLHL15, TRIM32, and the CRL3-gigaxonin-USP15 pathway provide functional clues related to neuronal-structure maintenance and protein clearance [113,114,115]. At this stage, the main value of these findings is that they provide functional endpoints that can be tracked. A readout is more likely to become a useful indicator linking gene abnormalities to disease phenotypes only if it changes consistently with pathogenic variants and recovers in parallel when the corresponding mechanism is corrected, rather than merely reflecting an accompanying alteration.

Omics and functional assays therefore serve primarily to screen and validate, rather than simply to expand the list of candidate molecules. Changes in protein abundance, ubiquitination, or molecular interactions can help identify targets worth further investigation, but they are not sufficient to establish a direct E3–substrate relationship. Stronger evidence requires stepwise alignment among molecular interaction, E3-dependent ubiquitination or protein stability, variant-specific effects, and functional rescue. Existing *UBE3A* ligase activity assays can directly evaluate ligase function [130], endogenous activity biosensors can track intracellular *UBE3A* activity [131], and cell-based variant assessment can compare the functional consequences of different variants [132]. Paternal *UBE3A* reporter systems, including human pluripotent stem cell reporters and dual-reporter mouse models, can also be used to monitor silencing and reactivation [133,134]. The value of these tools lies in narrowing the candidate space and determining which molecular changes can form stable, reproducible, and rescue-correlated readouts, thereby providing a basis for prioritizing future targets.

## 5. Conclusions and Future Perspectives

E3-related NDDs have expanded from a small number of classic syndromes into a broader group of neurodevelopmental disorders involving different E3 ligases, E3-complex components, substrate-recognition factors, and other ubiquitination-associated regulatory proteins. Although these genes differ in inheritance pattern, variant type, and molecular consequence, existing genetic and functional studies still indicate a degree of mechanistic convergence: the associated abnormalities may affect different levels of neurodevelopment, synaptic and network maturation, and cellular homeostasis, and GO enrichment further supports this convergence from a functional-annotation perspective. At the same time, these mechanisms differ markedly in their proximity to therapeutic translation. *UBE3A*-related Angelman syndrome has developed a relatively clear path involving *UBE3A* restoration and paternal allele reactivation. Some downstream mechanisms involving *CUL3*, *CUL4B*, and *KCTD13* have begun to show preclinical modulatory evidence, whereas many candidate substrates and functional readouts still require further validation of their direct regulatory relationships, reproducibility, and disease relevance.

E3-associated NDDs should not be treated as a single pathogenic entity. Distinct E3 ligases can converge on similar clinical phenotypes through different mechanisms, developmental windows, and cell types. Similar clinical phenotypes may arise from disruptions occurring at different developmental stages, cellular contexts, and molecular levels. A process-oriented perspective is therefore more informative than gene listing alone. Future studies should determine how specific variants alter E3 function and downstream regulation. These effects should be distinguished according to whether they involve loss of expression, reduced catalytic activity, altered substrate recognition, abnormal complex assembly, increased dosage, or gain- or change-of-function mechanisms.

The next phase of this field should focus on linking variant-specific molecular changes to reproducible disease-relevant readouts and functional rescue. Emerging human-relevant and spatially resolved approaches may help clarify when and where E3 dysfunction becomes pathogenic. For *UBE3A*-related AS, therapeutic development must optimize timing, delivery, brain-region distribution, expression dosage, and long-term safety. For other E3-related genes, the priority is to establish reproducible variant-specific assays and substrate-validation pipelines. Ultimately, progress will depend on linking genetic variation to specific molecular consequences while distinguishing validated mechanisms from secondary downstream changes.

## Data Availability

No new data were created or analyzed in this study. Data sharing is not applicable to this article.

## Abbreviations

The following abbreviations are used in this manuscript:

NDD	Neurodevelopmental disorder
NDDs	Neurodevelopmental disorders
DD	Developmental delay
ID	Intellectual disability
ASD	Autism spectrum disorder
AS	Angelman syndrome
Dup15q	15q11-q13 duplication syndrome
UPS	Ubiquitin-proteasome system
Ub	Ubiquitin
E1	Ubiquitin-activating enzyme
E2	Ubiquitin-conjugating enzyme
E3	Ubiquitin ligase
HECT	Homologous to the E6AP carboxyl terminus
RING	Really interesting new gene
CRL	Cullin-RING ligase
CRL3	*CUL3*-based Cullin-RING ligase complex
CRL4B	*CUL4B*-based Cullin-RING ligase complex
BTB	Broad-complex, Tramtrack, and Bric-a-brac
DCAF	DDB1- and CUL4-associated factor
NPC	Neural progenitor cell
CNV	Copy number variant
UPD	Uniparental disomy
*UBE3A*-ATS	*UBE3A* antisense transcript
E/I	Excitatory/inhibitory
iPSC	Induced pluripotent stem cell
hiPSC	Human induced pluripotent stem cell
RNA-seq	RNA sequencing
WES	Whole-exome sequencing
WGS	Whole-genome sequencing
